# Microwave ablation and portal vein embolization for staged hepatectomy

**DOI:** 10.1093/jscr/rjaf564

**Published:** 2025-07-30

**Authors:** Alexandra Simbeck, Tim Reese, Karl J Oldhafer

**Affiliations:** Department for Surgery, Asklepios Barmbek, Rübenkamp 220, 22307 Hamburg, Germany; HPB Surgery, Karolinska University Hospital, Stockholm, Sweden; Department for Surgery, Asklepios Barmbek, Rübenkamp 220, 22307 Hamburg, Germany

**Keywords:** colorectal liver metastasis, staged hepatectomy, ALPPS, microwave ablation

## Abstract

Associating liver partition and portal vein ligation for staged hepatectomy (ALPPS) enables resection of extensive colorectal liver metastases (CRLM) by inducing rapid hypertrophy of the future liver remnant (FLR). However, the classical approach is often associated with significant morbidity, particularly in patients with compromised liver parenchyma. We present a case utilizing the associating microwave ablation and portal vein ligation for staged hepatectomy (AMAPS) technique as a modified ALPPS procedure in a patient with chemotherapy-induced liver injury and bilobar CRLM.

## Introduction

Complete resection of colorectal liver metastases (CRLM) remains the gold standard, for achieving long-term survival. However, extensive hepatectomy is often limited by the necessity to preserve a future liver remnant (FLR) adequate in volume and function to avoid postoperative liver failure (PLF) [1, 2], particularly in cases of steatohepatitis, cirrhosis, or chemotherapy-related liver injury [3, 4].

One method of inducing hypertrophy of the FLR is associating liver partition with portal vein ligation for staged hepatectomy (ALPPS).

When the original ALPPS procedure was proposed by Schnitzbauer *et al.* in 2012, it was associated with high morbidity and mortality rates [5].

Since then, the technique has been refined with various modified forms of ALPPS, such as partial ALPPS or hybrid ALPPS, which have been successful in minimizing the most common risks associated with the procedure [6].

Another such modification is RALPP (Radio-frequency-assisted Liver Partition with Portal Vein Ligation) [7] and its later variation AMAPS (associating microwave ablation and portal vein ligation for staged hepatectomy) [8].

With both techniques, in-situ splitting of the liver during ALPPS Step I is replaced with a ‘virtual split’ using ablative techniques, which are otherwise often applied in patients with larger tumors (3–5 cm) and advanced liver disease [9].

Here, we present a case where we used the AMAPS technique to perform ALPPS Step I in a patient with colorectal liver metastasis.

## Case presentation

**Figure 1:**
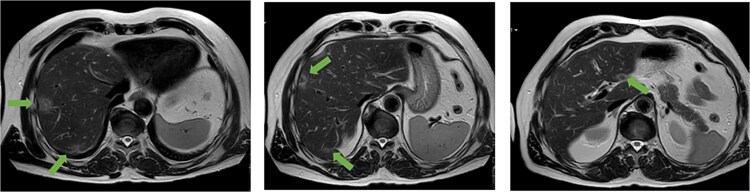
Axial MRI slices obtained preoperatively showing bilobar colorectal liver metastases. Key lesions are marked with arrows.

A 56-year-old man, who had undergone anterior rectal resection for upper rectal carcinoma 9 months prior, presented with ˃20 synchronous liver metastases involving both hepatic lobes (Fig. 1).

The patient had been treated with 12 cycles of FOLFOX postoperatively. Recent scans showed a favorable chemotherapy response and no signs of extrahepatic disease, so we decided to proceed with a two-stage hepatectomy concept.

### Step I

First, a right subcostal laparotomy with a vertical extension to the xiphoid process was performed. Abdominal exploration revealed no signs of extrahepatic metastasis, and in particular, there was no indication of local tumor relapse or peritoneal carcinomatosis.

However, further examination revealed drug-induced liver injury indicative of ‘blue liver syndrome.’

Intraoperative sonography showed three metastases in the left lateral liver, as well as five more in segment IV. More than 10 metastases were found within the right liver.

First, a standard cholecystectomy was performed. The liver parenchyma already showed a considerable bleeding tendency, prompting intermittent application of the Pringle maneuver (42 min total) during metastases resection. All three left lateral metastases were resected using an ultrasonic surgical aspirator combined with an electrothermal bipolar tissue sealing system. Next, four metastases in segment IV were excised via wedge resection. Lastly, microwave ablation was performed on the remaining metastasis in segment IVa.

Due to the continuously increased bleeding tendency, we decided to forego a traditional ‘in-situ-split’ in favor of an ‘ablation split’ using microwave coagulation. During this, ablation was simultaneously performed on two metastases in segment V located close to the future resection margin, *thereby allowing preservation of the maximum amount of liver tissue possible.* In total, five overlapping ablations using 100 W for 3 min each were performed.

Perioperative blood loss was 800 ml; blood transfusion was not required.

Postoperatively, the patient developed biliary leakage ISGLS grade A, but his recovery was otherwise uneventful.

Right portal vein embolization (PVE) was performed 5 weeks postoperatively, with the pre-interventional FLR being 34.5% at the time.

### Step II

The patient underwent the second operation 10 weeks after stage I, at which point FLR had increased to 39.5%, and a LiMAx test indicated normal liver function (371 μg/kg/h).

**Figure 2:**
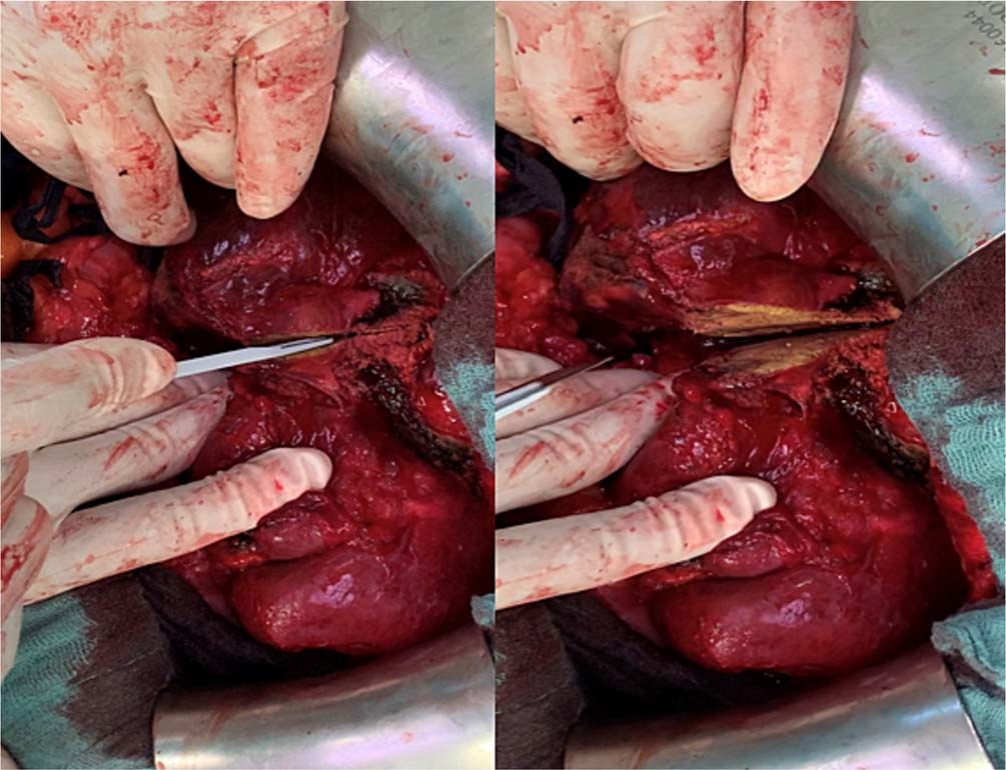
Parenchymal transection of the liver following prior MWA demarcation.

Due to adhesions and the patient’s once again increased bleeding tendency, mobilization of the right liver was difficult. After careful exposure of the liver hilum, the right hepatic artery and the right portal vein were severed. Along the region previously marked via microwave ablation, parenchymal resection could now be performed without any bleeding using only a scalpel (Fig. 2).

The remaining resection was carried out using the combination of ultrasonic aspirator and bipolar tissue sealing system as previously described. Another intraoperative ultrasonography showed no further metastatic lesions in the remaining liver parenchyma. Intraoperative blood loss was ⁓1500 ml, due to diffuse bleeding during liver mobilization. The patient was extubated in the operating room before being transferred to the ICU, where he spent 3 days before returning to the regular surgical ward for routine postoperative care. The postoperative LiMAx test performed on the 1st postoperative day again indicated satisfactory liver function (141 μg/kg/h).

After step II surgery, the patient received transfusion of two erythrocyte concentrates during postoperative recovery.

He again developed postoperative biliary leakage ISGLS grade A but experienced no further complications and was discharged on the 8th postoperative day.

**Figure 3:**
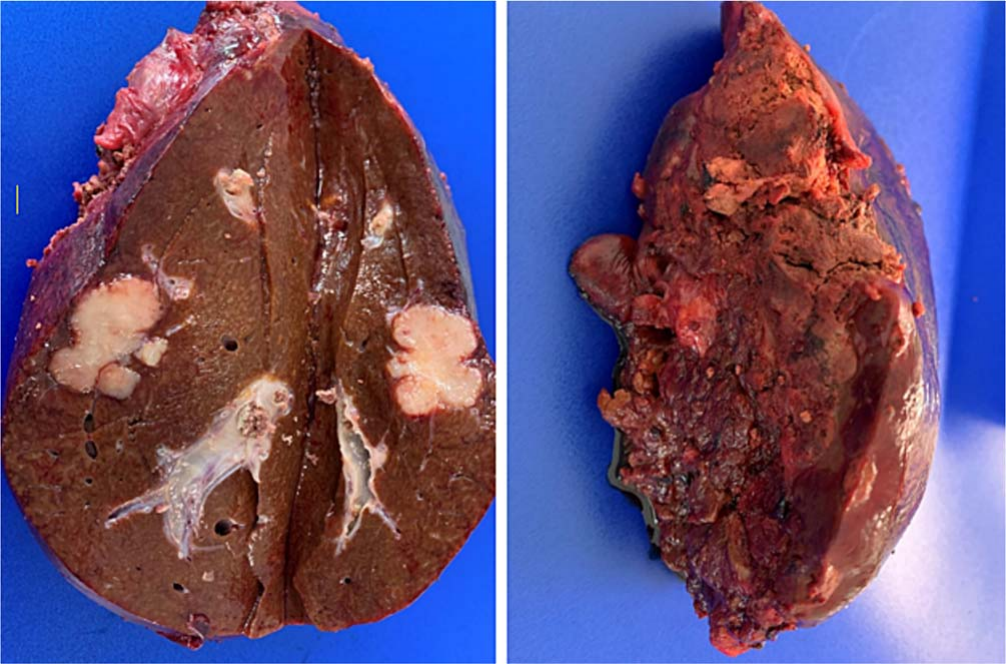
Post-resection specimen of the right hepatic lobe (segments V–VIII) showing multiple metastases from colorectal carcinoma.

Pathological analysis of the surgical specimen later revealed that R0 resection had been achieved [full TNM classification for the cancer of the upper rectum was pT3 pN1b (3/21) pM1a (HEP)] (Fig. 3).

After sufficient recovery, he received adjuvant chemotherapy consisting of another three cycles of FOLFOX.

## Discussion

Although ALPPS is a popular method for achieving rapid hypertrophy of the FLR, the procedure’s high morbidity and mortality rates require particularly rigorous patient selection.

The RALPPS modification has previously been shown to reduce common serious complications of traditional ALPPS, such as severe postoperative biliary leakage, abdominal infection, and post-hepatectomy liver failure [10, 11].

Similar to radiofrequency ablation, microwave thermal ablation/coagulation (MWA) is an established technique used to treat primary and metastatic hepatic tumors by inducing coagulative necrosis [12].

The combination of MWA and PVE offers the option of safer liver resection along the avascular plane [13, 14].

In this specific case, a traditional in-situ split during step I would not have been feasible due to the patient’s fragile liver tissue and excessive bleeding tendency. Moreover, multiple metastases were located in segment V, some directly adjacent to the future resection margin, posing a risk of tumor re-infiltration to the FLR during the waiting period between Step I and II.

Performing the MWA split allowed us to control intraoperative bleeding while simultaneously clearing the relevant resection line of tumor infiltration without sacrificing more tissue than necessary.
